# The EvMed Assessment: A test for measuring student understanding of core concepts in evolutionary medicine

**DOI:** 10.1093/emph/eoad028

**Published:** 2023-08-30

**Authors:** Taya Misheva, Randolph M Nesse, Daniel Z Grunspan, Sara E Brownell

**Affiliations:** Research for Inclusive STEM Education Center, School of Life Sciences, Arizona State University, Tempe, AZ, USA; Center for Evolutionary Medicine, School of Life Sciences, Arizona State University, Tempe, AZ, USA; Department of Integrative Biology, College of Biological Science, University of Guelph, Guelph, ON, Canada; Research for Inclusive STEM Education Center, School of Life Sciences, Arizona State University, Tempe, AZ, USA

**Keywords:** assessment, education, instructor resource, core principles

## Abstract

**Background and objectives:**

Universities throughout the USA increasingly offer undergraduate courses in evolutionary medicine (EvMed), which creates a need for pedagogical resources. Several resources offer course content (e.g. textbooks) and a previous study identified EvMed core principles to help instructors set learning goals. However, assessment tools are not yet available. In this study, we address this need by developing an assessment that measures students’ ability to apply EvMed core principles to various health-related scenarios.

**Methodology:**

The *EvMed Assessment (EMA)* consists of questions containing a short description of a health-related scenario followed by several likely/unlikely items. We evaluated the assessment’s validity and reliability using a variety of qualitative (expert reviews and student interviews) and quantitative (Cronbach’s α and classical test theory) methods. We iteratively revised the assessment through several rounds of validation. We then administered the assessment to undergraduates in EvMed and Evolution courses at multiple institutions.

**Results:**

We used results from the pilot to create the *EMA* final draft. After conducting quantitative validation, we deleted items that failed to meet performance criteria and revised items that exhibited borderline performance. The final version of the *EMA* consists of six core questions containing 25 items, and five supplemental questions containing 20 items.

**Conclusions and implications:**

The *EMA* is a pedagogical tool supported by a wide range of validation evidence. Instructors can use it as a pre/post measure of student learning in an EvMed course to inform curriculum revision, or as a test bank to draw upon when developing in-class assessments, quizzes or exams.

## Introduction

Evolutionary medicine (EvMed) is a relatively new field that applies an evolutionary lens to deepen our understanding of health and disease [1, 2]. Given its potential to influence medicine and biomedical research, there have been calls to include EvMed in pre-medical education [1]. Efforts to answer this call can be seen in the growing number of courses teaching EvMed at the postsecondary level [6, 7]. A majority of research-intensive universities in the USA now offer at least one undergraduate course that either focuses on EvMed exclusively or includes EvMed as a unit within a more general evolution or health-related curriculum [6]. These courses are typically housed within biology or anthropology departments or cross-listed between the two [6]. The increasing prevalence of EvMed instruction at the undergraduate level has created a need for pedagogical resources, which has been partially addressed by a growing number of instructor resources including textbooks [8], lesson plans or activities [9, 10] and journal articles that include insights for teaching EvMed [3, 11]. While these resources focus on *what* to teach in EvMed or *how* to teach EvMed, resources that help assess student understanding of EvMed are largely absent. To fill this gap, we developed and validated the EvMed Assessment (EMA), 45 true-or-false items distributed across 11 question sets.

### Assessment and core principles in backward design

The EvMed Assessment was developed as part of a larger effort to assemble pedagogical resources for EvMed using backward design [9]. Backward design is an educational method that stresses the importance of alignment among curricular components [12–14]. With backward design, the instructor first specifies the learning goals, then develops assessments to evaluate mastery of the learning goals, and lastly develops instructional materials that match the assessments and learning goals. The key benefit of this approach is that it facilitates internal alignment of what students are taught, what is assessed, and which concepts the instructor deems most important [15].

To provide EvMed instructors with a resource for setting learning goals, our team (D.G., R.N. and S.B.) previously conducted a study to identify the theoretical principles that underlie EvMed. In that study, we iteratively surveyed 56 biologists, anthropologists, medical doctors and other researchers who contributed to the field of EvMed via publications and participation in the International Society for Evolution, Medicine and Public Health [16]. This survey yielded a list of 14 core principles of EvMed: (i) types of explanation (proximate vs ultimate causes), (ii) evolutionary processes, (iii) reproductive success, (iv) sexual selection, (v) constrains on adaptation, (vi) general evolutionary trade-offs, (vii) life history theory, (viii) levels of selection, (ix) phylogeny, (x) coevolution, (xi) developmental plasticity, (xii) immune defenses, (xiii) environmental mismatch and (xiv) and the impact of cultural practices on health and disease [16]. The inclusion of each core principle on this list was approved by at least 80% of the panellists. Agreement with the inclusion of each principle ranged from 80% for sexual selection to 100% for types of analyses, evolutionary processes, cultural practices, and life history theory.

These core principles provide guidance for EvMed instructors as they construct course learning goals [15] and course assessments. However, creating effective assessment questions can be time consuming and difficult, making questions that have been tested and validated across multiple contexts a useful resource. Creating this type of resource that aligns with the core principles for use by EvMed instructors was our goal in developing the EMA.

### Assessments with validation evidence

Assessment validity is the extent to which the assessment measures what it claims to measure. More specifically, it is the extent to which inferences drawn from assessment scores accurately reflect students’ actual understanding of the relevant material. Assessments with validation evidence have been rigorously tested to demonstrate that various extraneous factors—such as unclear wording, questionable scientific accuracy of the content, or student use of general test-taking skills—appear to have minimal influence on the scores [17–19]. Two common types of assessments with validation evidence are concept inventories [21] and programmatic assessments [22–24]. Concept inventories are designed to measure students’ understanding of a relatively narrow scientific idea, such as natural selection [20, 25], genetic drift [21] or macroevolution [26]. Alternatively, programmatic assessments have been designed for an entire field, such as ecology and evolution [22], physiology [24] and general biology [23]. They typically measure a range of core concepts that have been established by a group of disciplinary experts (e.g. *Vision and Change* report on undergraduate biology education) and are not limited to one particular course; rather, they assess student learning within a field throughout an entire undergraduate program. Both concept inventories and programmatic assessments aim to produce aggregate results that enable inferences to be made about a curriculum’s efficacy and collective student learning.

While assessments with validation evidence share structural similarities with instructor-generated course exams, these assessment types differ in their primary purpose and design process. Instructor-generated exams usually lack validation evidence and cover a portion of the course, which may or may not be based on specific learning goals [27]. These primarily measure individual students’ learning to provide a summative assessment (i.e. a grade), but are not always effective at measuring collective student learning, which is a primary function of concept inventories and programmatic assessments. This additional function requires higher standards of validity and reliability [17, 28].

Validity and reliability are frameworks for evaluating the quality of the inferences that can be drawn from a measurement tool, such as an assessment or survey [17]. Validity addresses the question of whether an assessment truly measures what researchers want it to measure. This is typically evaluated using both quantitative and qualitative methods that examine (i) the extent to which the assessment accurately represents the relevant knowledge domain, (ii) the extent to which it requires students to use the skills and thought processes that researchers want them to use and (iii) whether assessment items that are designed to measure the same concept get answered the same way [17–19, 29]. Reliability addresses the consistency of an assessment’s measurements. This is typically evaluated using quantitative methods that look at test–retest consistency across individual students or at the consistency of students’ answers across items within a single assessment [17–19, 29]. Creating an exam with this level of validation is time consuming, and thus not practical for typical instructor-generated exams.

Many of the EvMed core principles are general evolutionary concepts that are covered within existing concept inventories; for example, understanding of phylogeny can be measured using the Evolutionary Tree Concept Inventory [30], and understanding of coevolution can be measured using the Host-Pathogen Interactions Concept Inventory [31]. (see Supplementary Table 1 for a list of concept inventories that cover various EvMed core principles [20, 21, 26, 30–34]). These existing concept inventories can be used to measure student understanding of individual evolutionary concepts within various non-human contexts. However, the application of evolution to novel contexts in human health and disease may influence how students operationalize these concepts, so there is a need for a new EvMed-specific assessment.

### Project goals

Our goal was to provide EvMed instructors with an assessment resource with validation evidence that uses questions at high levels on Bloom’s taxonomy [3, 35] to measure student understanding of the EvMed core principles by requiring students to apply the core principles to a range of novel health-related scenarios. The design of the EMA is similar to a programmatic assessment because it covers multiple core concepts within a field, rather than a single relatively narrow idea. However, unlike a programmatic assessment that is typically administered across multiple courses in an undergraduate program, we foresee the use of the EMA being more similar to a concept inventory, which are primarily used in individual courses. We acquired validity evidence for this assessment using expert feedback, student interviews, and by administering the assessment in 11 different EvMed courses in universities throughout the USA.

## Development

### Assessment format and question development

**Figure 1.**
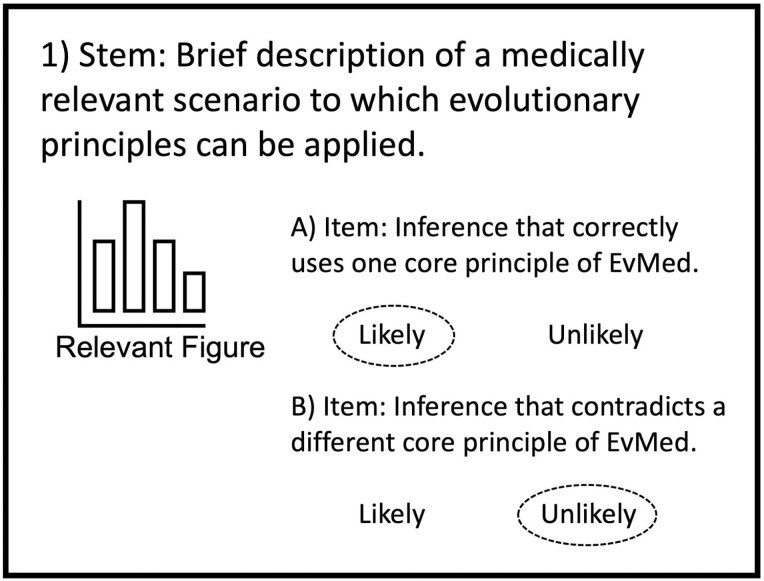
Question format on the EvMed Assessment

The EMA uses a modified version of the multiple-true-false (MTF) format, in which each question contains an informational premise (stem) followed by several true-or-false statements (items) (Fig. 1) [22–24]. The advantages of the MTF format over the standard multiple-choice format are that (i) students can answer more items in a set amount of time, (ii) the assessment can be used to explore student understanding of multiple principles applied to the same scenario, and (iii) the MTF format has been shown to mimic free-response reasoning by revealing individual misconceptions within a chain of reasoning [21, 23, 36]. We modified the MTF format by replacing the options of ‘true’ and ‘false’ with ‘likely’ and ‘unlikely’ [22, 24]. This helped accommodate the inherent uncertainty in applying evolutionary principles to health-related topics and made it easier to create items that were scientifically accurate and sufficiently difficult. This approach reflects the tentative nature of science [37–39] and has previously been used in evolution and ecology assessment [22].

The question stems describe a scenario in one to two paragraphs, often accompanied by a table or figure. These scenarios cover a wide range of health-related topics, including genetic conditions, cancer, infectious disease, ageing and mental health. We included a variety of health-related topics to reflect the broad scope of EvMed as a discipline [1, 3, 4]. For each stem, we wrote items that present a single inference or prediction based on information in the stem and test competencies in one or more core principles. While each item was intended to primarily assess one core principle, inherent conceptual overlap led to some items covering additional closely related principles (e.g. life history theory and trade-offs) [16]. The entire set of items within a question covers several core principles that can be applied to the given scenario. To ensure that items assess conceptual understanding rather than knowledge or familiarity with technical terms, we limited the use of scientific and medical jargon.

**Figure 2.**
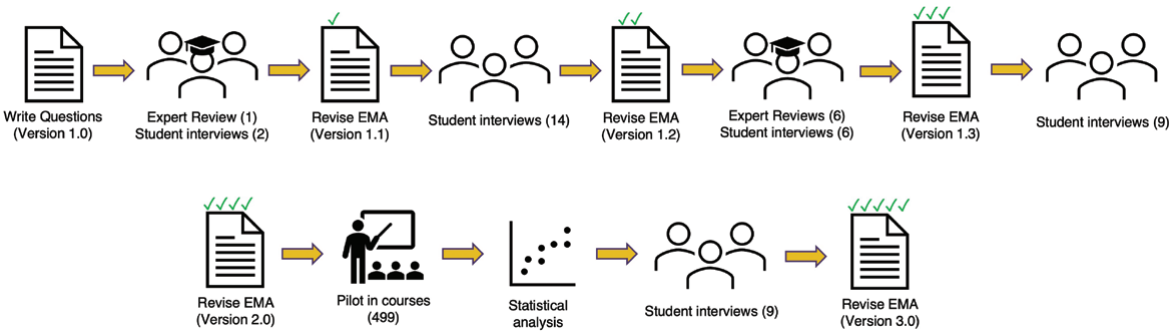
Development and validation process of the EvMed Assessment. Parenthetical numbers indicate sample size

To develop the first draft of the EvMed Assessment (version 1.0), D.G. and T.M. wrote questions based on the core principles of EvMed [16]. Both researchers had extensive prior teaching and coursework experience in EvMed and general familiarity with the EvMed literature. We wrote stems by condensing information from a wide range of sources and generating hypothetical scenarios based on well-studied phenomena. This is a standard approach to question development for concept inventories that require students to transfer conceptual knowledge to novel scenarios [21–23]. R.N. and S.B. provided feedback on questions. R.N. is an international expert in EvMed, having written several books on the field, and S.B. is an expert in assessment development in biology education. Version 1.0 of the assessment included a total of 70 likely/unlikely items across 14 question stems (Fig. 2). This version included items covering every principle except sexual selection, where we found it impossible to identify examples of evolutionary inferences that could be conveyed with a brief description, had a clear-cut answer [39], and did not resort to gender essentialism.

### Qualitative validation: version 1.0 to 2.0

Version 1.0 was revised by a process of iterative evaluation and improvement of its content and substantive validity based on multiple rounds of student interviews and expert reviews that addressed issues identified with individual questions and items. See Fig. 2 for an overview of the entire assessment development process. The Institutional Review Board of Arizona State University approved the procedures for this study (ASU IRB #00010655).

Content validity addresses whether the assessment offers an accurate representation of the relevant knowledge domain; it is typically evaluated using expert review [17–19, 29]. To assess content validity, seven scholars active in teaching or conducting research in EvMed reviewed the assessment for accuracy on two separate occasions. A single expert (R.N.) reviewed the initial draft of the assessment (version 1.0). The other six experts (one PhD student, four postdoctoral scholars and one Assistant Professor) reviewed the updated version 1.2. The reviewers were asked to (i) point out any information that is inaccurate or presented as being more scientifically settled than it really is, (ii) select the correct answer for each item and rate their confidence in their answer on a scale of 1–10 and (iii) provide any other concerns they have (e.g. clarity, difficulty level, etc.). We revised or deleted items if at least one reviewer answered ‘incorrectly’ with high confidence or deemed the item overly complex or debatable. We also addressed any noted issues with accuracy or clarity as recommended by the reviewers.

Substantive validity addresses whether students are using the skills and thought processes that the developers want them to use when answering items on an assessment [17–19, 29]. This involves checking for issues like students answering correctly despite using incorrect reasoning or answering incorrectly despite using correct reasoning (e.g. because they misinterpreted what the item was asking). To assess substantive validity, we conducted a total of 31 think-aloud interviews with undergraduate students on versions 1.0–1.3 of the assessment. During interviews, students read the question stem out loud and explained the reasoning behind their answer choices. The interviewer took notes and asked clarifying questions about the students’ reasoning. The interviewer noted if the student (i) interpreted the stems and items correctly, (ii) was able to select the correct answer despite using the wrong reasoning or (iii) selected the wrong answer despite using the correct reasoning. We were watchful for the latter two issues because an effective assessment needs to consistently differentiate between correct use of the concepts being tested versus the presence of misconceptions [22–24].

The 31 think-aloud interviews were distributed across four initial rounds of revision (Fig. 2). In order to obtain a sample that reflects the student population for which this assessment is intended, we recruited participants who were currently enrolled in an EvMed course at one institution and recruited late in the semester to ensure they had some level of content knowledge. All interviews were conducted by either T.M. or D.G. and audio recorded. We revised an item or stem any time two or more students displayed the same issue, such as when the use of a particular misconception led to the correct answer, or when multiple students were able to answer an item using only common knowledge without engaging with core concepts. This process resulted in version 2.0 of the assessment, with 14 questions containing 62 items across all questions (Fig. 2).

### Quantitative validation of version 2.0

#### Data collection and processing

To quantitatively evaluate the psychometric properties of the assessment, we conducted a large-scale pilot test of version 2.0. Given the length and cognitive complexity of the assessment, we were concerned about the potential effect of survey fatigue on student attentiveness [22, 40]. To keep testing time under the recommended 30-min time limit for most respondents [22, 40], we reduced the length of the assessment by dividing version 2.0 into seven core questions and seven supplemental questions. When taking the assessment, all students received the seven core questions along with a random sample of five supplemental questions. The core questions were chosen by identifying the minimum number of questions needed to cover all the EvMed core principles. To do so, we categorized items by the principles they tested and mapped out which question sets would provide coverage over an appropriate range of principles while also presenting a variety of health topics.

Version 2.0 of the assessment was administered online as an out-of-class assignment to 732 undergraduate students across seven different courses at six institutions (Table 1). The assessment was accompanied by a demographic survey. Four of the participating courses administered the assessment in a single semester, two administered it in two consecutive semesters, and one administered it in three consecutive semesters. Thus, the assessment was administered to students in 11 total course offerings; 10 of these were EvMed courses and one was an evolution course (Table 1). We administered the assessment toward the end of term so that the sample population would reflect the target population for the assessment. However, targeting EvMed courses made it difficult to obtain a large sample size because EvMed courses tend to be relatively small. To increase our sample size and evaluate how the assessment performs with students who have a background in evolution, but not EvMed, we also administered the assessment in one high-enrolment evolution course. Participants received a small amount of extra credit based on completion.

**Table 1.** Overview of courses that administered version 2.0 of the EvMed Assessment

Course	University	Number of semesters	Total students	Final student sample
EvMed	Non-selective R1 HSI; southwest	1	21	16
Evolution	Non-selective R1 HSI; southwest	1	305	179
EvMed	Non-selective R1; mountain west	2	70	54
EvMed	Non-selective M1; southeast	2	32	25
EvMed	Very selective R1; west coast	1	45	31
EvMed	Selective R1; southeast	1	13	11
EvMed	Selective R1; west coast	3	246	183

Hispanic-serving institution (HSI)

Only students who took at least 10 min. to complete the entire assessment were included in the final sample.

Of the 732 students who completed the assessment, 684 (93.4%) consented to their data being used for the study. Because this was a low-stakes assignment, we were concerned that some students may have put in minimal effort (e.g. quickly clicked through the questions). Thus, we inspected how long students took to complete the entire survey and determined that a minimum of 10 min appeared to be an appropriate cut-off, which is consistent with the cut-offs used in similar studies [22, 23]. This eliminated 184 total students from our sample. One additional student was removed because they left most items blank, leaving 499 students (68.3% of initial population) in our final sample. The majority of students in this sample identified as women (76.4%), with a smaller percent of men (21.6%) and non-binary students (2%). Most students identified as White/European American (45.6%), Asian/Asian American (22.8%) or Hispanic/Latinx (12.6%) (see Table 2 for full demographics).

**Table 2.** Demographics of students who took part in think-aloud interviews and the pilot study

Student characteristic	Interviews (*N* = 39)		Quantitative validation (*N* = 499)	
	*N*	%	*N*	%
Gender				
Men	10	25.6%	108	21.6%
Women	29	74.4%	382	76.4%
Nonbinary	0	0%	10	2%
Race/Ethnicity				
Asian/Asian America	9	23.1%	114	22.8%
Black/African America	3	7.7%	18	3.6%
Hispanic/Latinx	11	28.2%	63	12.6%
Native American/Native Hawaiian	1	2.6%	5	1%
White	15	38.4%	228	45.6%
Other	0	0%	72	14.4%
Academic year				
Lower (first year/sophomore)	22	56.4%	140	28%
Upper (junior/senior)	15	38.5%	353	70.6%
Graduate	2	5.1%	5	1%
Major				
Biology-related fields	20	64.5%	n/a	n/a
Anthropology-related fields	5	16.1%	n/a	n/a
Other	6	19.4%	n/a	n/a
Missing	8	20.5%	n/a	n/a
Highest parental education level				
Completed bachelor’s degree	29	76.3%	n/a	n/a
Did not complete bachelor’s degree	9	23.6%	n/a	n/a
First language				
English	28	71.8%	n/a	n/a
Not English	11	28.2%	n/a	n/a

not applicable (n/a)

Interview sample includes 31 students who participated in the initial qualitative validation (version 1.0–1.3) and 8 who participated in additional post-pilot validation (version 2.0).

#### Statistical analyses

We used classical test theory (CTT) to examine the psychometric properties of the assessment. CTT is a theoretical framework that is widely used to assess educational and psychological measurement tools, including their overall validity and reliability, as well as the performance of individual items within an assessment [41, 42]. While other frameworks such as item response theory (IRT) have more recently been developed, CTT remains appropriate to use when the sample size does not meet the recommended minimum for IRT [23, 24].

#### Assessment-level reliability and validity

An assessment’s reliability is based on the consistency of its measurements. One form of reliability is internal consistency, which describes the extent to which all the items in a test measure the same concept. We used Cronbach’s α to measure the internal consistency of version 2.0. For assessments that aim to measure a single construct, internal consistency is considered ‘excellent’ when α > 0.9, ‘good’ when 0.8 > α > 0.9 and ‘acceptable’ when 0.7 > α > 0.8 [43]. Cronbach’s α was 0.71 for the entire assessment and 0.73 for the core questions alone. Given that the EMA measures understanding of multiple concepts, this is an acceptable level of internal consistency.

An assessment’s validity is based on how well the scores reflect the construct of interest. One form of validity is criterion validity, which is based on the idea that test scores should correlate with variables theorized to be associated with the construct being measured. To evaluate criterion validity, we compared students’ performance on the assessment to their year of study and whether they were enrolled in an evolution course or an EvMed course. Test scores were higher for students who were further along in their educational career and likely had a stronger evolution background. A small sample of first-year students who did particularly well on the assessment are a notable exception, although they may be exceptional students as evidenced by their enrolment in upper-level courses. Further, we found that students enrolled in EvMed courses scored higher (*M* = 22.38, SD = 4.53) than students enrolled in the evolution course (*M* = 19.84, SD = 4.34) (*t*(389) = 6.13, *P* < 0.001). This provides a level of criterion validity that this test is measuring students’ EvMed knowledge (Table 3).

**Table 3.** Mean student scores on the EvMed Assessment, listed according to academic year and course enrolment

	Percent correct on core	Percent correct on core + supplement
Year in college		
First (*n* = 8)	65.6 (6.25)	65.2 (5.45)
Second (*n* = 132)	63.5 (1.19)	62.0 (1.03)
Third (*n* = 136)	67.3 (1.30)	66.5 (1.07)
Fourth (*n* = 195)	69.0 (0.99)	67.1 (0.87)
Fifth + undergraduate (*n* = 22)	72.6 (2.72)	71.5 (2.34)
Graduate student (*n* = 5)	83.1 (3.64)	78.3 (4.13)
Course		
EvMed (*n* = 320)	70.2 (0.79)	68.7 (0.67)
Evolution (*n* = 179)	62.2 (1.01)	60.9 (0.86)

Standard errors are in parentheses. One students did not provide their academic year.

#### Item-level statistics

Validity and reliability are properties of the assessment as a whole. However, it is also important to analyse the performance of individual items. To do this, we examined item difficulty and item discrimination. Item difficulty (*P*) is the percent of students who answered the item correctly; it ranges from 0.0 (everyone answered incorrectly) to 1.0 (everyone answered correctly). While there are no standard cut-off values for item difficulty, some recommend values between 0.3 and 0.7 [44]. Item discrimination (*D*) is a measure of how well an item distinguishes between students with high test scores versus those with low test scores. Discrimination scores range from −1.0 to 1.0; values near 1.0 indicate that mainly high-scoring students answered the item correctly, values near 0.0 indicate that high-scoring and low-scoring students performed equally, and negative values indicate that low-scoring students were more likely to answer correctly [44]. Discrimination scores above 0.2 are recommended [45]. Using this cut-off value, we flagged items with a *D* of <0.2 for further review, as these items are potentially problematic due to insufficient differentiation. A total of 29 out of 62 items were flagged. Meanwhile, items that had both good difficulty scores (0.3–0.7) and good discrimination scores (≥0.2) were deemed suitable for inclusion in version 3.0 without any additional review.

### Final draft of the assessment (version 3.0)

#### Creating version 3.0

We began our review of the 29 flagged items by examining each item’s discrimination score, difficulty score, think-aloud interview results and expert feedback from the previous validation stage. We deleted two questions because a majority of items within these questions were flagged for low discrimination, which indicates that these questions were performing poorly as a whole. Additionally, we deleted a third full question and three individual items from other questions because expert reviewers had expressed mild concern about their overall quality. We initially kept these in version 2.0 to further assess their performance, but deleted them after they were flagged for low discrimination. In total, we deleted eight flagged items in this manner.

Conversely, we kept seven flagged items as-is because (i) their discrimination scores were moderately below the recommended cut-off (*D* ≥ 0.13), (ii) their difficulty scores were within the recommended range and (iii) prior student interviews suggested no validity issues. Based on prior interviews, most of these items appear to display somewhat low discrimination because they test principles for which student misconceptions are particularly widespread.

The remaining 14 flagged items were revised to improve clarity and then re-assessed for substantive validity via additional student interviews. We conducted eight additional interviews with students recruited from an upper-level biology course; all students had taken at least one upper-level course on evolution, but no courses on EvMed specifically (see Table 2 for demographics). Based on these interviews, we deleted one item and kept the remaining 13 items for the final version of the assessment.

#### Overview of version 3.0

The final version of the EvMed Assessment consists of six core questions and five supplemental questions; each question contains 3–6 items. The six Core Questions present scenarios within six different health-related topics: genetic conditions, autoimmune disorders, infectious disease, cancer, mental health and drug function. Items in the core questions cover all of the EvMed core principles at least once, with the exception of sexual selection (Supplementary Table 2).

The supplemental questions were designated as such because they (i) address core principles that already have coverage in the core questions and (ii) cover health-related topics that are either already covered in the core questions (e.g. infectious disease) or are less central to EvMed (e.g. zoology). All supplemental questions passed the validity checks and can be added into the EvMed Assessment as one sees fit. A complete copy of the EvMed Assessment can be found in the Supplementary Material.

## Discussion

### Using the EvMed Assessment

The EMA is a multi-purpose assessment tool. The EMA can be used to (i) gather data on student learning to improve instruction, (ii) identify which core principles students find most difficult, (iii) draw from this pool of questions with validity evidence when designing in-class activities or course exams and (iv) compare the efficacy of different teaching settings or styles. When administered at the very beginning of a course, the EMA can be used to identify which core principles are least familiar to students and adjust instruction for that term accordingly. When administered at the end of a course, the EMA can be used to identify which core principles students continue to struggle with and revise future iterations of the course to address those principles more effectively. Furthermore, instructors can also use the EMA as a test bank of high Bloom’s level questions with validation evidence, which can be used for formative assessments (e.g. worksheets or clicker questions) and summative assessments (e.g. exams). When using the EMA as a test bank, we encourage instructors to pick and choose content from both the core questions and supplemental questions to match the learning goals of the lesson or unit.

### Limitations and future directions

One limitation of the validation process was that most of the expert reviews were conducted by early-career scientists who may not have the same depth of knowledge as more senior researchers. This limitation, combined with the fact that EvMed as a discipline continues to evolve, means that instructors may disagree about whether some assessment items have settled, clear-cut answers. We encourage instructors to leverage their own expertise and the latest EvMed research when making judgement calls about whether to include all items when administering this assessment.

One limitation of the development process of this assessment stems from the fact that education research in EvMed is a relatively new area of inquiry [3–5, 6, 16], and we did not have an extensive literature base on student misconceptions about EvMed core principles to draw upon when designing this assessment. For this reason, we relied on general misconceptions about evolution [46] and our own observations during teaching and learning. However, future research into student misconceptions would be helpful in further development of EvMed education resources. This exercise would be particularly useful for principles that are uniquely important to EvMed and where student understanding has not been systematically investigated, such as evolutionary mismatch or trade-offs. This type of work could lay the foundation for more specific concept inventories in EvMed. We encourage other researchers to build on our current work by developing and refining additional assessment resources for EvMed.

## Supplementary Material

eoad028_suppl_Supplementary_Material
