# Supplementary material for: The EvMed Assessment: A test for measuring student understanding of core concepts in evolutionary medicine
Source: Evol Med Public Health. 2023 Aug 30;11(1):353–62. doi: 10.1093/emph/eoad028 (PMC10597536; doi:10.1093/emph/eoad028)
Supplement: eoad028_suppl_Supplementary_Material [file eoad028_suppl_supplementary_material.docx]

**SUPPLEMENTAL TABLES AND FIGURES**

| **Supplemental Table 1.** Concept inventories that cover one or more core principles of evolutionary medicine. C.I. stands for concept inventory. | | | |
| --- | --- | --- | --- |
| **Title** | **Citation** | **Description** | **Relevant EvMed core principle(s)** |
| Evolutionary Tree C.I. (ETCI) | Kummer et al. (2019) | 24 items; multiple choice. Population: bio majors.  Contact authors for copy. | Phylogeny |
| Phylogeny Assessment Tool (PhAT) | Smith et al. (2013) | 3 items; free response.  Population: bio majors.  Available in manuscript. | Phylogeny |
| C.I. of Natural Selection (CINS) | Anderson et al. (2002) | 20 items; multiple choice.  Population: bio majors, non-majors.  Available in supplement. | Evolutionary processes |
| Concept Assessment of Natural Selection (CANS) | Kalinowski et al. (2016) | 24 items; multiple choice. Population: bio majors.  Available in supplement. | Evolutionary processes |
| Genetic Drift Inventory (GeDI) | Price et al. (2014) | 22 items; agree/disagree.  Population: bio majors.  Available in supplement. | Evolutionary processes |
| Measure of Understanding of Macroevolution (MUM) | Nadelson & Southerland (2009) | 27 multiple choice items, 1 free-response. Population: bio majors, non-majors. Available in appendix. | Evolutionary processes; phylogeny |
| Host-Pathogen Interactions C.I. | Marbach-Ad et al. (2009) | 18 items; multiple choice. Population: microbiology students. Contact authors for copy. | Coevolution |
| Evo-Devo C.I. | Perez et al. (2013) | 11 items; multiple choice. Population: bio majors.  Available in supplement. | Adaptive constraints; developmental plasticity;  trade-offs |

| **Supplemental Table 2**. Assessment coverage of the EvMed core principles. Items are listed according to the core principles that they each test. Some items test two or more closely related core principles. There are no items on sexual selection. | | |
| --- | --- | --- |
| **Core Principle** | **Items (Core)** | **Items (Supplemental)** |
| Types of explanation | 3D |  |
| Evolutionary processes | 1C, 3C, 4A, 4B, 4D, 4E, 4F | 8A, 9A, 9B, 9C, 9D, 9E, 10A, 10D, 11A |
| Reproductive success | 1A, 1B, 5A | 7D, 9A, 9B |
| Sexual selection | - | - |
| Constraints on natural selection | 5B | - |
| Trade-offs | 2B, 2C, 4C | 7A, 7B, 7C, 7D, 9A, 9B, 11C |
| Life history theory | 1A, 1B | 7A, 7B, 10A, 10B, 10D |
| Multiple levels of selection | 4A, 4B, 4D, 4E, 4F | - |
| Phylogeny | 2E, 6A | 10C, 10D |
| Coevolution | 6B, 6C | - |
| Plasticity | 5D | 8A, 8B, 8C |
| Defenses | 3A, 3B, 3C, 5A, 5B, 5C, 5D, 6C | - |
| Mismatch | 5D, 6C | - |
| Cultural Practices | 2A | 11C |
| General Biology | 2D, 3C | 1D, 11B |

**EVMED ASSESSMENT – CORE QUESTIONS**

**1.)** Consider the following two genetic disorders:

*Huntington’s chorea*

Huntington’s chorea is a rare genetic disorder that is characterized by depression, forgetfulness, involuntary movements, and slurred speech. Symptoms of Huntington’s start appearing between the ages of 30 and 50, and typically worsen over time. Huntington’s leads to death about 15 - 20 years after the onset of symptoms. The disorder is caused by mutations found in a gene called the Huntington gene. Huntington’s afflicts 0.1% of individuals in Europe, with rates tending to be even lower among populations of non-European ancestry. However, one place where rates of Huntington’s chorea are higher than in populations of European descent is the village of Barranquitas in Venezuela, where the percent of individuals with Huntington’s is closer to 10%. Huntington’s chorea is caused by novel mutations only 10% of the time.

*Tay-Sachs disease*

Tay-Sachs disease is a rare genetic disorder. Approximately 0.4% of individuals are carriers of Tay-Sachs, but this rate is closer to 4% in Ashkenazi Jewish, French Canadian, and Louisiana Cajun populations. Most individuals born with Tay Sachs die within the first 4 years of their life.

Using the information above, evaluate the following statements as either likely or unlikely.

1A.) Because individuals die due to the disease, the prevalence of Huntington’s chorea will inevitably reach zero in Barranquitas.

Likely (0) **Unlikely (1)**

1B.) Natural selection is a stronger evolutionary force on Tay-Sachs than on Huntington’s chorea.

**Likely (1)** Unlikely (0)

1C.) Natural selection is the best explanation for the high rate of Huntington’s chorea in the Venezuelan population.

Likely (0) **Unlikely (1)**

1D.) All humans have a copy of the Huntington gene in their genome. [SUPPLEMENTAL ITEM]

**Likely (1)** Unlikely (0)

**2.)** Virus A is a seasonal airborne virus that is more common in the fall than at other times of the year. While this virus is responsible for many deaths each year, most healthy individuals are usually able to clear the virus after a couple of weeks. These individuals are then immune to the specific strain they were infected with for the rest of their lives. However, humans are exposed to new strains of this virus every year.

2A.) A single vaccination during childhood would provide effective lifelong protection against Virus A.

Likely (0) **Unlikely (1)**

2B.) Imagine a strain of Virus A emerges that kills its host 100% of the time, but is otherwise similar to past strains. This new strain would be more likely to spread if it killed its host quickly after initial infection.

Likely (0) **Unlikely (1)**

2C.) Imagine a strain of Virus A emerges that kills its host 100% of the time, but is otherwise similar to past strains. This new strain would be more likely to persist in a city with 6 million people compared to a small rural population.

**Likely (1)** Unlikely (0)

2D.) An antibiotic would be an effective treatment against Virus A.

Likely (0) **Unlikely (1)**

**
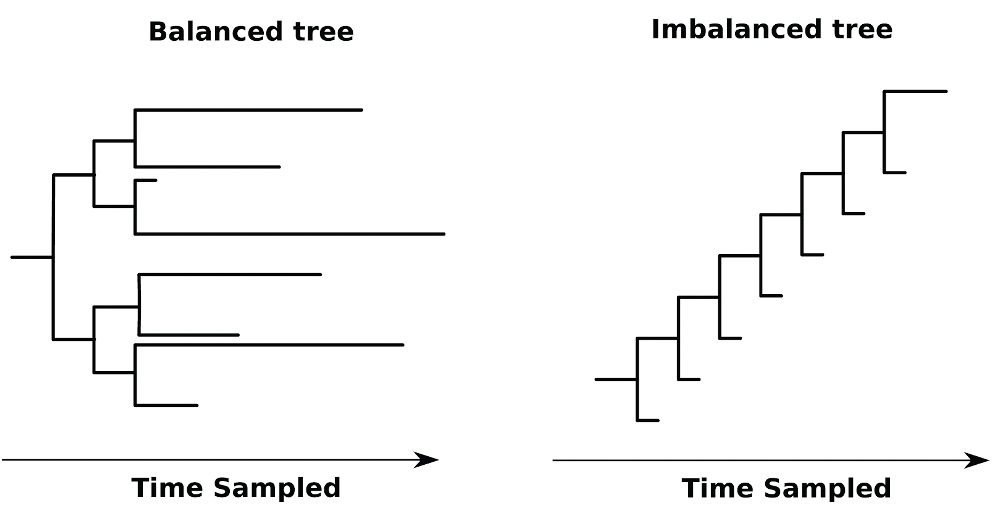
**

2E.) Researchers took samples of Virus A each year over the past 30 years from sick individuals. When they reconstruct a phylogeny from these viral samples, it would more closely resemble an imbalanced tree than a balanced tree

**Likely (1)** Unlikely (0)

**3.)** Many infectious illnesses are accompanied by a fever. Some drugs, such as ibuprofen, are designed to reduce fever. Having a fever contributes to the sensation of being sick, so many people take fever-reducing drugs in order to feel better. A woman decides to take ibuprofen because she currently has a fever due to having the flu.

Using the information above, evaluate the following statements as either likely or unlikely.

3A.) The ibuprofen will slow down the rate at which the flu virus replicates.

Likely (0) **Unlikely (1)**

3B.) The ibuprofen will help fight off the flu virus.

Likely (0) **Unlikely (1)**

3C.) The ibuprofen will lead to drug resistant flu viruses.

Likely (0) **Unlikely (1)**

3D.) Discovering drugs like ibuprofen relies on research that uncovers evolutionary (ultimate) explanations rather than research that uncovers mechanistic (proximate) explanations.

Likely (0) **Unlikely (1)**

**4.)** Chemotherapy is used to kill cancerous tumor cells. Historically, chemotherapy is administered in high doses aimed to eliminate all of the cells within a tumor. Tumors shrink in response to chemotherapy, but most often they eventually grow back because the cancer cells become resistant to the chemotherapy drug.

A researcher proposes a new way to administer chemotherapy, which they call adaptive therapy. They suggest using occasional doses of chemotherapy with the goal of killing most, but not all, of the tumor cells. They say that an issue with constant high dosage chemotherapy is that it leaves chemotherapy-resistant tumor cells without chemotherapy-sensitive cells to compete with for growth.

The figure below shows a model of a tumor over time, where traditional high dosage chemotherapy is started at time 6. Each circle represents a cancer cell. White cells are sensitive to chemotherapy, while darker cells show greater resistance to chemotherapy.

*Using the information above, evaluate the following statements as either likely or unlikely.*


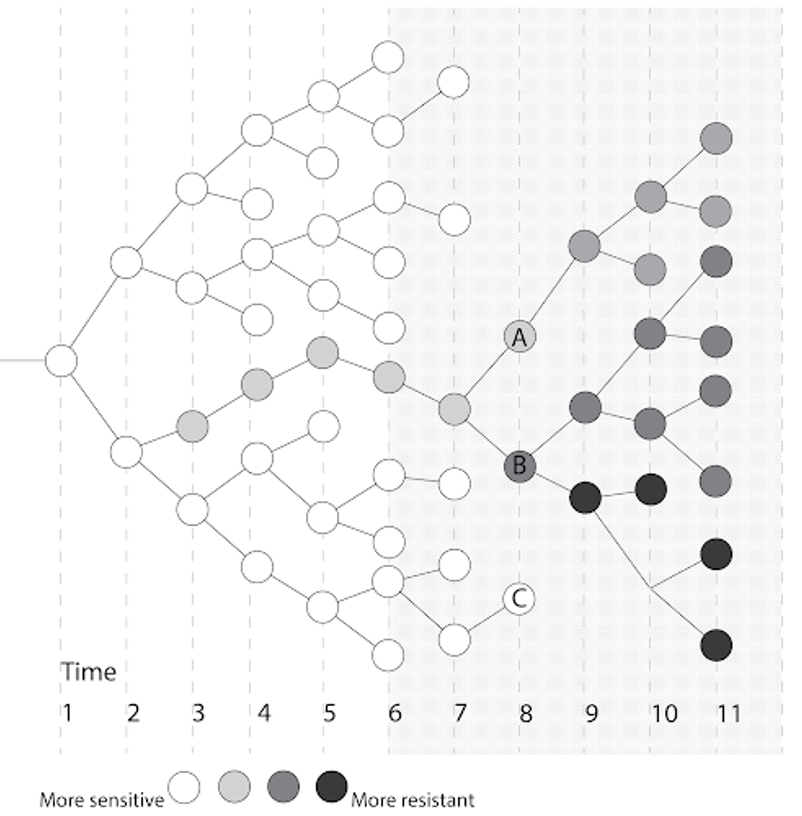


4A.) Tumors become chemotherapy resistant because each cancer cell individually develops resistance to chemotherapy over time.

Likely (0) **Unlikely (1)**

4B.) Assume chemotherapy was stopped between times 7 and 8 as part of an adaptive therapy treatment. For this to successfully prevent the development of a chemotherapy resistant tumor, cell C would have to outcompete cells A and B.

**Likely (1)** Unlikely (0)

4C.) In the absence of chemotherapy, chemotherapy-resistant cancer cells replicate faster than chemotherapy-sensitive cells.

Likely (0) **Unlikely (1)**

4D.) The high dosage chemotherapy would have been more effective at completely eliminating the tumor if it started at time 4.

Likely (0) **Unlikely (1)**

4E.) Adaptive therapy would be effective if the first round of chemotherapy that starts at time 6 was stopped at time 10.

Likely (0) **Unlikely (1)**

4F) The chemotherapy-resistant cancer cells had no selective advantage over the non-resistant cancer cells before chemotherapy was started.

**Likely (1)** Unlikely (0)

**5.)** Humans vary in the frequency and severity of anxiety they experience. Some people have generalized anxiety disorders (GAD), which are characterized as excessive anxiety and worry about various events or activities. Most people experience a moderate amount of anxiety, while some experience no anxiety at all. Over the past several decades, the number of individuals with GAD has increased.

5A.) Individuals who never experience anxiety have higher fitness than those who experience anxiety in moderate amounts.

Likely (0) **Unlikely (1)**

5B.) Generalized anxiety disorder is a useful adaptation.

Likely (0) **Unlikely (1)**

5C.) Theoretically, natural selection will result in disorders of too much anxiety more often than disorders of too little anxiety.

**Likely (1)** Unlikely (0)

5D.) The increase in the number of individuals with generalized anxiety disorder over the past several decades is being driven by natural selection.

Likely (0) **Unlikely (1)**

**6.)** The phylogeny below shows the relationship between four mammal species – A, B, C, and D. Species A is commonly infected with Worm 1, Species C is commonly infected with Worm 2, and Species D is commonly infected with Worm 3. In these three mammal species, individuals have chronic worm infections that typically start shortly after birth. Species B does not experience worm infections in the wild; it lives in a region that is not known to have any worms.

A zoo established captive breeding populations of Species A and B. All animals in the initial captive population of Species A were infected with Worm 1. The initial captive population of Species B entered the zoo without any infected individuals.

Shortly after the captive populations were established, all of the Species B individuals became infected with Worm 1. In response, the zoo de-wormed all captive animals of Species A and B. The animals were all adults when they were de-wormed. All future generations never experienced worm infections.


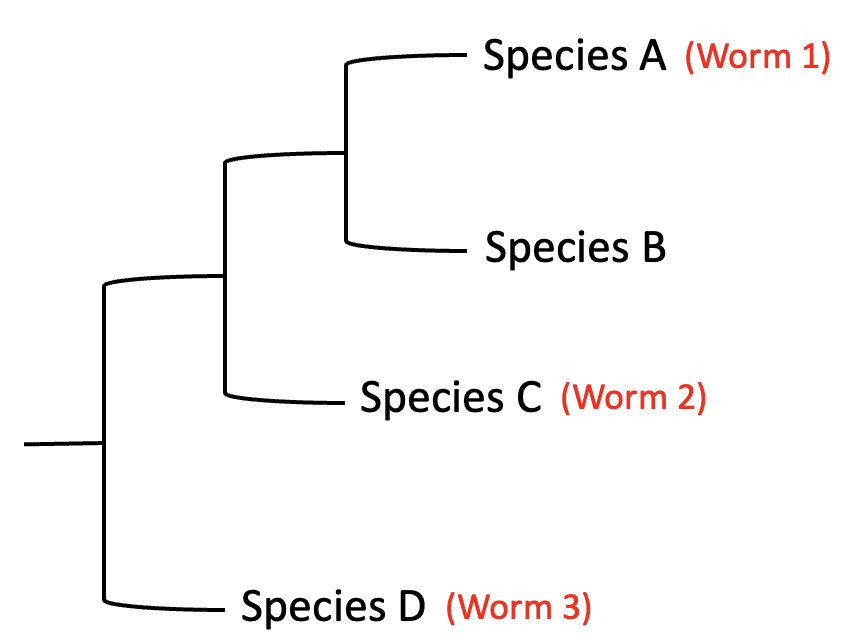


6A.) The last common ancestor of the four species experienced infections with Worm 3.

Likely (0) **Unlikely (1)**

6B) The symptoms of infection with Worm 1 are more harmful for Species B than Species A.

**Likely (1)** Unlikely (0)

6C) Species B is more likely than Species A to experience allergies in the zoo.

Likely (0) **Unlikely (1)**

**EVMED ASSESSMENT – SUPPLEMENTAL QUESTIONS**

**7.)** The Daf-2 gene in nematode worms is thought to play some kind of role in energy allocation. Researchers designed an experiment that more closely examines the function of the Daf-2 gene in *C. elegans*, a species of nematode worm. First, the researchers created a strain of worms that had a mutation in their Daf-2 gene. They found that the mutant worms had a longer lifespan than wild type worms without the mutation.

The researchers then tracked two populations of worms over time; one population started out with 50 Daf-2 mutant worms, and the other population started out with 50 wild type worms. After 8 generations, the researchers counted the number of worms in each population. The individual worms at the end of the experiment were not the same ones as at the start; all of them had been born during the course of the experiment. The results of this research are below.

|  | Number of worms: start of experiment | Number of worms: end of experiment | Average lifespan |
| --- | --- | --- | --- |
| Wild Type | 50 | 84 | 14 days |
| Mutant | 50 | 3 | 27 days |

7A.) Nematodes with the *Daf-2* mutation probably allocate less energy to reproduction than nematodes without the mutation.

**Likely (1)** Unlikely (0)

7B.) Nematodes with the *Daf-2* mutation probably allocate less energy to somatic repair than nematodes without the mutation.

Likely (0) **Unlikely (1)**

7C.) *Daf-2* influences two or more phenotypic traits.

**Likely (1)** Unlikely (0)

7D.) The *Daf-2* mutation that results in longer lifespan increases the fitness of nematodes.

Likely (0) **Unlikely (1)**

**8.)** The average height of individuals from two different human populations between 1930 and 2010 is shown below. Population A is an equatorial population living in a relatively industrialized country. People in population A have access to good sanitation and a wide variety of nutritious food. Very little migration has occurred into or out of Population B over the past 80 years, although many health initiatives have taken place along with increased industrialization.

Individuals from Population A also have much darker skin color than those from Population B. Skin color can be measured via melanin index (MI), where a higher MI indicates darker skin, while a lower MI indicates less melanin and lighter skin. Most people in Population A have melanin indices of 50 - 60; while most people in Population B have melanin indices of 20 - 30.

From 1935-1940, thousands of families immigrated from Population B to Population A. Many immigrant couples had children after arriving in Population A.


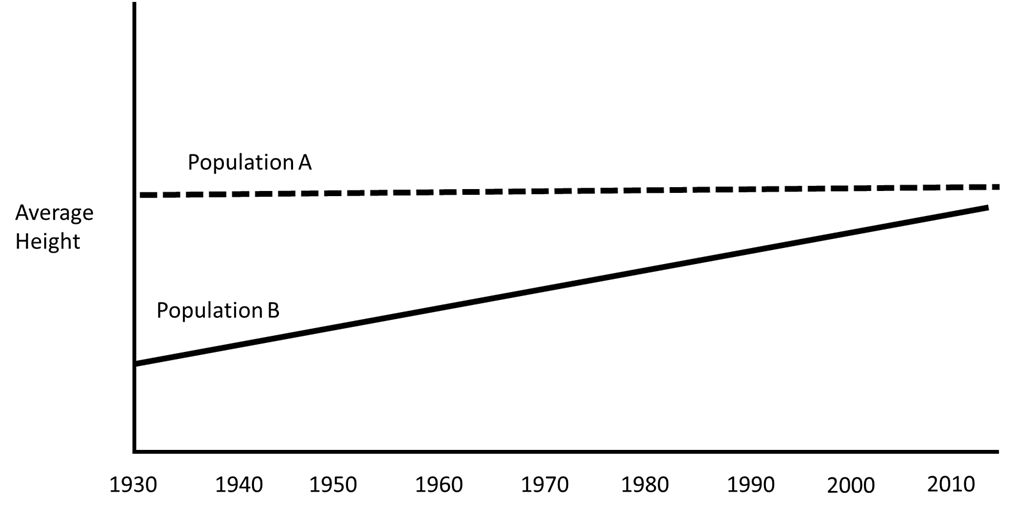


**
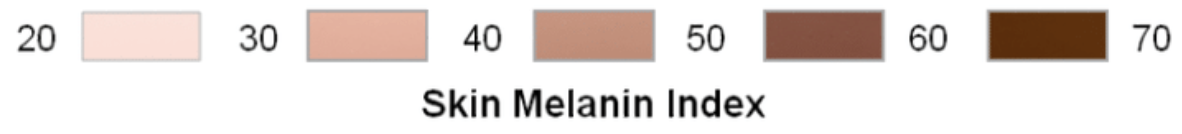
**

Source: Wilson et al. (2011) [66].

8A.) The graph above suggests that height is undergoing natural selection in population B.

Likely (0) **Unlikely (1)**

8B.) Children born in Population A to Population B immigrant parents during the 1940s will grow up to be taller, over average, than their same-age peers in Population B.

**Likely (1)** Unlikely (0)

8C.) Children born in Population A to Population B immigrant parents will have melanin indices closer to 55 than 25.

Likely (0) **Unlikely (1)**

**9.)** A parasite endemic to Island A causes a deadly illness in humans. This parasite is transmitted between human hosts by a species of fly, and is among the most common causes of death for individuals aged 10-45. The fly that carries the parasite is not found on Island B.

Humans have the Z gene. People who have at least one copy of the H1 allele of gene Z are resistant to the disease-causing parasite. However, those who are homozygous for the H1 allele are at high risk of developing Z-associated kidney disease, which leads to an early death. The “wild type” (WT) allele of gene Z does not protect the person from the disease-causing parasite; however, the WT allele also is not associated with kidney disease.

An allele that is not found on either island is H5*. Individuals homozygous for H5* are resistant to the parasite, while carriers of H5* do not experience increased risks of kidney disease.

| **Table 2.** Relationship between genotype, disease risk, and parasite susceptibility. | | |
| --- | --- | --- |
|  | **Risk of Gene-Associated Chronic Kidney Disease** | **Susceptibility to Parasite** |
| WT/WT | None | Susceptible |
| WT/H1 | None | Resistant |
| H1/H1 | High | Resistant |
| WT/H5* | None | Susceptible |
| H5*/H5* | None | Resistant |

| **Table 1.** Gene Z allele frequencies between islands. | | |
| --- | --- | --- |
|  | **Island A** | **Island B** |
| Parasite | Common | Absent |
| WT allele frequency | 0.78 | 0.85 |
| H1 allele frequency | 0.22 | 0.15 |
| H5* allele frequency | 0.00 | 0.00 |

9A.) If the parasites from Island A were eliminated, the frequency of the H1 allele would decrease over time.

**Likely (1)** Unlikely (0)

9B.) If a drug were administered to the population on Island A that cured chronic kidney disease with no side-effects, the frequency of the H1 allele would decrease over time.

Likely (0) **Unlikely (1)**

9C.) Assuming the islands have the same population size, a novel H5* allele is more likely to arise via mutation on Island A than on Island B.

Likely (0) **Unlikely (1)**

9D.) If two teenage brothers, each with WT/H5* genotypes, immigrated to Island A, there would immediately be strong selection for the H5* allele leading to a higher H5* allele frequency over time.

Likely (0) **Unlikely (1)**

9E.) If the allelic frequency of H5* on Island A was 0.2, it would face stronger positive selection if its effect on parasite resistance was dominant as opposed to recessive.

**Likely (1)** Unlikely (0)

**10.)** Imagine two species, Species A and Species B. These species diverged from one another 20 million years ago. Species A is larger than Species B. Individuals of Species A die of old age at around 40 years, while individuals of Species B die of old age at around 20 years. One of the two species faces higher levels of predation than the other.

10A.) Earlier natural death in Species B evolved as an adaptation that enables offspring to have access to resources and survive.

Likely (0) **Unlikely (1)**

10B.) Species A experiences lower levels of predation compared to Species B.

**Likely (1)** Unlikely (0)

10C.) Based on the information above, we can determine that the age of death in the common ancestor of Species A and Species B is around 30 years of age.

Likely (0) **Unlikely (1)**

10D.) Species A would be predicted to have more novel defense mechanisms against cancer compared to Species B.

**Likely (1)** Unlikely (0)

**11.)** The Human Immunodeficiency Virus (HIV) infects and kills immune cells, which can lead to AIDS. AIDS is a very deadly condition that can develop in HIV-infected people of any age. The first recorded case of AIDS was in 1981, and researchers suspect that HIV first transferred to humans from other primates in the early 20th century.

Gene A influences the structure of immune cells. This gene has two alleles: A1 and A2. People who have a least one copy of the A1 allele have an average level of resistance against the flu virus, and are susceptible to HIV infection. People who have two copies of the A2 allele are highly resistant to HIV infection, but have weaker defenses against the flu virus. People who are homozygous for the A2 allele are 20% more likely to die from the flu after the age of 40.

In northern Europe, around 12% of people have at least one copy of the A2 allele, and a smaller proportion of people have two copies. The A2 allele is much more rare in the rest of the world, including southern Africa.

| **Table 1.** Relationship between genotype, HIV susceptibility, and flu susceptibility | | |
| --- | --- | --- |
| **Genotype** | **HIV Defense** | **Flu Defense** |
| A1/A1 | Susceptible | Normal |
| A1/A2 | Susceptible | Normal |
| A2/A2 | Immune | Lower, especially after age 40 |

| **Table 2.** A2 allele frequencies, HIV/AIDS prevalence, and flu prevalence across geographic regions. | | | |
| --- | --- | --- | --- |
| **Region** | **A2 Allele Frequency** | **Prevalence of HIV/AIDS** | **Prevalence of Flu** |
| Southern Africa | 3% | 15% of people are diagnosed | Common  (most encounter at least once in lifetime) |
| Northern Europe | 12% | 0.25% of people are diagnosed | Common  (most encounter at least once in lifetime) |

11A.) The relatively high frequency of the A2 allele in northern Europe is the result of selective pressure created by HIV/AIDS.

Likely (0) **Unlikely (1)**

11B.) The A2 allele was more adaptive in northern Europe than in southern Africa at some point before the 20th century, but is now probably more adaptive in southern Africa than in northern Europe.

**Likely (1)** Unlikely (0)

11C.) With genome-editing technology, it is possible to artificially introduce two copies of the A2 allele into a human embryo. Doing so is likely to benefit the health and longevity of a baby born in northern Europe.

Likely (0) **Unlikely (1)**
